# Investigation of non-National Immunization Program vaccination intentions in rural areas of China

**DOI:** 10.1186/s12889-023-16390-4

**Published:** 2023-08-04

**Authors:** Xiuli Wang, Yaru Fan, Wei Wang

**Affiliations:** 1 School of New Media, Peking University, Beijing, China; 2 School of Journalism and Communication, Hunan University, Changsha, Hunan Province China

**Keywords:** National immunization program, Vaccine awareness, Vaccination intention, Vaccination behavior, Rural area, China

## Abstract

**Background:**

China’s current immunization program was revised in 2007. Some common childhood vaccines such as those for influenza, pediatric pneumonia, Haemophilus influenzae, varicella, and rotavirus have not been included in the National Immunization Program (NIP) and need to be purchased by children’s guardians at their own expense. Rural areas, constrained by economic development and vaccine awareness, have a low non-NIP vaccination rate and more family medical expenses and social burden. This study aims to examine the awareness and attitude of rural parents about non-NIP vaccines and relevant factors influencing their vaccination intention to provide strategic suggestions for expanding and improving the Chinese government’s NIP policy.

**Methods:**

A qualitative method of in-depth interviews were conducted for this study. We interviewed 30 rural parents in a central Chinese village to investigate their awareness of non-NIP vaccines and their vaccination intention and behavior. All the interview data were analyzed through the Colaizzi seven-step data analysis method.

**Results:**

This study summarized the individual and social level factors influencing the non-NIP vaccination intention of rural parents. The individual level factors include four themes: perceived severity with physical harm, treatment consumption (cost of the treatment of the subject diseases), psychological burden, and social consequences being subthemes; perceived vulnerability with age vulnerability, medical history, immune quality (children’s underlying immune status), and environmental vulnerability (sanitary condition of the rural environment) as subthemes; perceived efficacy with effect perception, psychological comfort, protective strength, and functional compensation (functions of non-NIP vaccines unreplaceable by NIP vaccines) being subthemes; and perceived cost consisting of two subthemes cost burden and adverse reaction. The social level influencing factors include the vaccination opinions in rural social networks, the accessibility of health services and vaccine products, and the guidance and promotion of vaccination policies. These factors act outside of individuals’ subjective awareness and influence decisions regarding non-NIP vaccination in rural areas.

**Conclusion:**

Based on these influencing factors, this study constructs a structural model for non-NIP vaccination decision-making process in rural areas of China. The results play a guiding role in directing attention to children’s health, promoting non-NIP vaccination, facilitating the dissemination of vaccine knowledge in rural areas, and improving NIP policies and practices in China.

## Background

China established the National Immunization Program (NIP) in 1978, and Chinese children receive a total of 14 free vaccines since the expansion of the NIP in 2007. Despite the remarkable achievements of the NIP in the control of vaccine-preventable diseases (VPDs), some important vaccines still have not been included in China’s NIP to date and thus must be purchased by citizens at their own expense. The pneumococcal conjugate vaccine (PCV), the rotavirus and HPV vaccines are currently included in the immunization programs of more than 100 countries, excluding China. The Haemophilus influenzae type b (Hib) vaccine is included in immunization programs and available free of charge in 192 countries, accounting for 99% of the world’s countries; China is the only country in the WHO that does not include the Hib vaccine in its NIP.

Three main reasons may explain why these important vaccines have not been included in China’s immunization program. Firstly, the National Immunization Advisory Committee (NIAC), which was established in 2017 and is currently the national level government think tank to provide evidence-based NIP policy recommendations, and its precedent, Experts Advisory Committee on the Immunization Program (EACIP) established in 1982, has not yet recommended inclusion of new vaccines since 2007 due to the lack of formal working mechanism to update the formulary of NIP vaccines [1]. Although the EACIP played a very important role in advancing the national immunization schedule and promoting relevant laws and regulations, it was not tasked with recommending new vaccines to be introduced into the national Expanded Program on Immunization (EPI); while NIAC was designed to develop China’s immunization policy more from the structural and legal level, its operation faces many challenges such as lack of resources and limited capacity and means to monitor the safety, effectiveness and impact of vaccine recommendations [2]. Secondly, the financing for NIP expansion is limited, with insufficient and unsustainable funding both from central and local Chinese government. Thirdly, the lack of NIP workforce makes it difficult to support the expansion of immunization program with the number of NIP vaccinators per 10,000 people has decreased by 3.8% in 2013 and 1.7% in 2019 respectively [1].

Being excluded in the NIP, these vaccines can be received in China only at citizens’ own expense. As rural areas are economically underdeveloped with low per capita income and poor access to quality medical services for children, the non-NIP vaccination rate is high in economically developed urban areas of China but low in remote, rural areas, with as much as a 19-fold difference in the number of vaccinations across Chinese provinces [3]. In addition, China’s child mortality rate is significantly higher in rural areas than in urban areas, with the urban–rural ratios for the infant mortality rate and the under-five mortality rate reaching 1:1.94 and 1:2.29, respectively [4]. Taking Pneumococcal and Hib mortality as an example, an estimate of 49% of pneumococcal deaths and 67% of Hib deaths occurred in 2017 in China’s underdeveloped western region, which accounted for only 28% of the child population in that year [5]. Compared to the more developed eastern region, the western region had a higher estimated pneumococcal and hib mortality and a lower pneumococcal and hib vaccine coverage. The two non-NIP vaccine coverage varied greatly by region and province with Hib vaccine coverage being 50% or greater in economically developed areas and 2–7% in five underdeveloped western provinces [5]. Therefore, children in economically underdeveloped areas have a greater need for vaccines to reduce the VPDs and narrow down the death rate gap; promoting vaccination of rural children is critical, and expanding China’s NIP with new vaccines should be a priority of current health efforts.

Many studies have investigated vaccination intentions toward non-NIP vaccines and the influencing factors, and found that the dissemination of vaccine knowledge decreases fear and anxiety about vaccination and thus increases vaccination intention [6, 7]. Vaccine knowledge is closely related to the literacy level of children’s primary guardians, and the two together affect vaccination attitudes [8]. Media reports are common sources of information for parents to learn about vaccines [9], and negative vaccine incidents widely disseminated by the media may weaken public support for vaccines. Therefore, health education is needed to improve public awareness of vaccines [10, 11]. In addition, doctors [9], community healthcare workers [12], childhood vaccine providers (CVPs) [13], vaccinated groups [14, 15], and other sources of information also have impacts on vaccination intentions.

Existing studies on vaccination intention have mostly focused on individual vaccines. The aim of this study is to conduct an overall survey of non-NIP vaccination among children in rural areas of China and to investigate the factors influencing the intention to receive non-NIP vaccines by analyzing parents’ vaccine awareness and vaccination intentions and behaviors to provide strategic suggestions for expanding and improving the Chinese government’s NIP policy.

## Methods

This study used a qualitative in-depth interview method to collected original data from 30 rural parents in a village in Henan Province, China, and then carried out a Colaizzi seven-step data analysis under the guidance of a theoretical framework.

### Interviewees

The 30 rural parents recruited in this study were all from Duan Village, Xianglushan Township, Yiyang County, Luoyang City, Henan Province. Henan Province is located in the central region of China and is a representative agricultural province with a rural population accounting for approximately 9% of the total rural population in China [16], ranking first among the 34 provincial administrative regions. The survey area, Duan Village, has a total population of 3,952 people and 887 families, is located in the middle of Xianglushan Township, and has relatively convenient transportation access. The per capita income of Xianglushan Township residents was 16,597 yuan in 2020 compared to the per capita disposable income of 16,107.93 yuan for rural residents in Henan Province in the same year [17]. Therefore, the survey area, Duan Village, is consistent with and thus generally representative of the general situation of vast rural areas in China in terms of factors such as transportation, economic level, and population size.

A purposive sampling strategy was applied for practical and ethical reasons to select interviewees who met the research requirements. Parents who were not willing to participate in interviews and those who had never heard of non-NIP vaccines were not included in the sample. Considering that vaccination behaviors mostly involved children under 6 years old, the selected interviewees were all parents of children aged 0–6 years. In general, Chinese villages are managed by groups, and villagers of the same group are more closely related. A total of 12 groups were identified in Duan Village, and at least one family was selected from each group for the survey to ensure that the interviewees represented this village to the maximum extent.

The basic descriptive statistics of the interviewees were as follows. In terms of the age of the interviewees, most parents were aged between 20 and 40 years old, accounting for 90% (27) of the sample; parents aged 40 years old accounted for only 10%. Only-child, two-child, and three-or-more-child families accounted for 43%, 37%, and 20% of the study population, respectively. Among the interviewees, those with a junior high school education accounted for the largest proportion (53%); most of the interviewees were workers and farmers (63%), and more than 70% of the families had an annual income of less than 100,000 yuan, which is approximately consistent with the situation of ordinary rural families. Mothers accounted for more than 90% of the interviewed parents, as they usually assume more parenting responsibilities. Table 1 lists the detailed information for each interviewee. To protect the confidentiality of the participants, all identifiable information was replaced with a subject code (S01, S02, etc.).

**Table 1:** Interviewees’ demographic information

No	Age	Gender	Annual family income (approx.)	Number of children	Education level
S01	36	F	RMB50,000	3	Junior High School
S02	37	F	RMB80,000	4	Junior High School
S03	29	F	RMB50,000	1	University
S04	47	M	RMB70,000	4	Junior High School
S05	26	F	RMB80,000	1	Senior High School
S06	21	F	RMB10,000	1	Junior High School
S07	27	F	RMB50,000	2	Senior High School
S08	44	F	RMB10,000	2	Elementary school
S09	36	F	RMB30,000	3	Junior High School
S10	48	F	RMB50,000	1	Junior High School
S11	26	F	RMB20,000	2	Junior High School
S12	33	F	RMB60,000	2	Junior High School
S13	32	F	RMB100,000	3	Senior High School
S14	35	F	RMB100,000	1	Senior High School
S15	32	F	RMB50,000	2	College
S16	25	M	RMB50,000	2	Senior High School
S17	25	F	RMB40,000	1	College
S18	26	F	RMB150,000	1	University
S19	31	F	RMB90,000	2	Senior High School
S20	37	F	RMB40,000	2	Senior High School
S21	22	F	RMB100,000	1	Junior High School
S22	26	F	RMB50,000	1	Junior High School
S23	25	F	RMB70,000	1	College
S24	22	F	RMB100,000	1	Junior High School
S25	34	F	RMB200,000	3	Junior High School
S26	26	F	RMB100,000	2	Senior High School
S27	28	F	RMB100,000	1	Junior High School
S28	32	F	RMB100,000	2	Junior High School
S29	26	F	RMB70,000	1	Junior High School
S30	27	F	RMB50,000	2	Junior High School

### Interview implementation

A preliminary interview outline was established according to the framework of non-NIP vaccine awareness and vaccination attitudes, intentions, and behaviors based on classical health communication theories such as the knowledge-attitude-practice (KAP) model and the theory of planned behavior (TPB) [18] as well as related research on vaccine awareness, intentions, and influencing factors. Three parents of children aged 1, 2, and 6 years were contacted for a preinterview survey. The three interviewees were familiar with the concept of non-NIP vaccines, and all indicated that people around them typically receive non-NIP vaccines, indicating that Duan Village, as the selected research setting, was able to support the study of non-NIP vaccines. However, the three preinterviewees were unable to accurately name the non-NIP vaccines and the corresponding efficacies due to their limited knowledge.

Therefore, the researchers showed the Information Sheet on Common Non-NIP Vaccines for Children to the interviewees in the formal follow-up interview to facilitate answering relevant questions. In addition, in the preinterview, most of the children were found to have been vaccinated between 0 and 2 years of age. To make the study more targeted and facilitate the acquisition of more accurate and clear interview data, the interviewees for the formal survey were purposively screened such that parents with children aged 0–2 years were selected for interviews. Based on the results of the preinterview, the researchers also revised and improved the interview outline.

The semi-structured outline included vaccine awareness questions, such as “Which non-NIP vaccines do you know?” and “Do you think it is necessary for your child to receive non-NIP vaccines? Why?”; attitude/willingness questions, such as “Are you willing to have your child vaccinated for non-NIP vaccines?” and “Which vaccines are you planning to get vaccinated with? Why?”; and vaccination behavior questions, such as “Has your child ever received non-NIP vaccines?”, “Why did you have your child vaccinated?” together with other related questions, such as “From what sources do you get information about vaccines?” and “Who do you usually discuss vaccine issues with?”.

The researchers started the formal interviews on January 25, 2021, continuing through the Chinese New Year holiday. The interview process was conducted in conjunction with data compilation, and all interviews were completed on March 15, 2021. Before and during the Chinese New Year holiday, the researchers visited village groups as units, and the interviews were mainly conducted face to face, with an average duration of approximately 35 min. After the Chinese New Year holiday, some interviewees were out of town for work and were thus interviewed via telephone. Before the interview, the interviewees were presented with the Information Sheet on Common Non-NIP Vaccines for Children to help them express themselves accurately. Information such as the interviewee’s name, age, education level, and annual household income was collected at the end of the interview. The entire interview process was voice recorded after obtaining consent from each interviewee, and the recordings were transcribed into text in a timely manner after the interview.

### Data analysis

The text from the interviews was coded using the Colaizzi seven-step data analysis method [19]. After carefully reading the text and becoming familiar with the data (step 1), the researchers identified significant statements (step 2) to formulate repeated patterns of meaning based on a cluster analysis of keywords (step 3). Emerging common themes (factors influencing the intentions of rural parents regarding non-NIP vaccination) were summarized from clustered meanings (step 4) and were discussed and illustrated with relevant verbatim quotes from interviewees (step 5) to build a structural diagram of the non-NIP vaccination decision-making process of rural parents (step 6). Finally, three copies of the original interview data were retained to validate the fundamental structure (step 7).

## Results

This study analyzed the awareness of rural parents about non-NIP vaccines and relevant factors influencing their vaccination intention, followed by the construction of a theoretical model.

### Vaccine awareness and attitude

Individuals’ knowledge and awareness of non-NIP vaccines are central to their vaccination intention. Analysis of the interview data indicated that rural parents had some awareness of and highly recognized the necessity of the non-NIP vaccination.

Disease names affect vaccine awareness. Most interviewees had some knowledge of non-NIP vaccines. The most frequently mentioned non-NIP vaccine was that for hand, foot, and mouth (HFM) (*n* = 22), followed by those for varicella (*n* = 18), rotavirus/autumn diarrhea (*n* = 16), influenza (*n* = 10), meningitis (*n* = 8), pneumonia (*n* = 6), and HPV (*n* = 4). In contrast, the WHO-recommended Hib vaccine and vaccines for measles and mumps were rarely mentioned by rural parents. Interviewees generally expressed ignorance about the combined vaccines and the difference among different types of vaccines. The scientific names and English abbreviations caused cognitive difficulties for some rural parents and affected their identification of vaccine information. For example, some interviewees did not know that the EV71 vaccine is for HFM disease and that MM is the combined attenuated live vaccine for measles and mumps.

Evidently, rural parents mainly relied on the identification of common diseases to understand vaccines. The interviewees who mentioned the HPV vaccine all referred to “cervical cancer.” As interviewee S05 said, “The cervical cancer vaccine sounds very important; after all, it is cancer.” Intuitive and clear disease names provide a direct means for rural parents to identify non-NIP vaccines.

The necessity of non-NIP vaccination is highly recognized in rural areas*.* During the interview, 75% of the interviewees agreed that their children receiving the non-NIP vaccines was necessary and important. Among them, the older and more educated parents had higher awareness of the necessity of non-NIP vaccination. For the 25% of the interviewees who indicated that they were unwilling to participate in vaccination or thought that such vaccines were unnecessary, many parents began to develop a positive inclination after the researchers detailed the efficacy of non-NIP vaccines and the diseases that could be prevented. Therefore, the reluctance of rural parents for non-NIP vaccination is largely due to a lack of knowledge about relevant vaccines, further confirming the importance of strengthening health education and knowledge popularization to improve vaccine awareness.

### Sources of vaccine information

Rural parents’ non-NIP vaccination information originates from multiple channels, including medical personnel, friends/relatives, the internet and social media, and offline health courses. Among them, medical personnel were the most frequently mentioned and the main source of vaccine information for rural parents, with an occurrence frequency of 66.7%, indicating the high influence of medical workers on rural parents. Close social ties in rural areas also make friends/relatives an important source of information, with an occurrence frequency of 53%. The internet and social media channels were more commonly referred to by interviewees as the app “Xiaodoumiao,” a mobile vaccination information platform launched by the Henan Provincial Center for Disease Control and Prevention. The app allows scheduling appointments, provides access to vaccination records, offers reminders for vaccinations and precautions, and contains various popular science publications on vaccines. The offline health courses mentioned by the interviewees referred to the public welfare program “Mummy Class” offered at a local county hospital—a series of vaccine health and training courses for new mothers at Yiyang County Hospital of Luoyang City, Henan Province, where local medical staff regularly give lectures on the functions, precautions, and vaccination conditions of common vaccines using offline multimedia software. Vaccine training courses have a positive impact on vaccine awareness. Parents who attended the “Mummy Class” courses generally not only had a better understanding of non-NIP vaccines in terms of the basic knowledge of vaccine functions, vaccination conditions, precautions, side effects, and vaccine manufacturers but also had a higher intention to vaccinate than other interviewees who did not participate in the courses.

### Factors influencing vaccination intention from the individual level

Protection motivation theory [20, 21] proposes that people develop coping patterns—generating protective intentions and protective behaviors—through threat appraisal and coping appraisal. Threat appraisal refers to assessing the severity of a threatening event and consists of both the severity and vulnerability of the situation, while coping appraisal is how one responds to a threat and consists of response efficacy, self-efficacy, and response costs. Drawing on protection motivation theory, the researchers conducted a Colaizzi analysis of the interview data to perform thematic clustering and found four factors that influence non-NIP vaccination intentions: perceived severity, perceived vulnerability, perceived efficacy, and perceived cost (see examples of extracted themes in Table 2).

**Table 2:** Examples of extracted themes

Step 2: Significant statement	Step 3: Formulation of meaning	Step 4: Theme clustering	Factors
S03 “There is a picture showing a child who was healthy at first and then infected with disease. It is too scary.”	Considerable physical harm and clinical consequences caused by disease	Physical harm	Perceived severity
S25 “Being sick can bring a huge burden to my kid and my whole family. Therefore, I would rather spend money on this paid vaccine for my child.”	High cost of disease treatment	Treatment consumption	
S13 “My child suffered from autumn diarrhea, and we as parents felt distressed for the little one.”	Psychological stress caused by disease	Psychological burden	
S09 “[Vaccination] prevented meningitis and helped my child to attend school. Otherwise, I would have worried that the teachers and the school would complain, and eventually, I had no choice.”	Family and school life impacted by disease	Social consequences	
S17 “Children are particularly prone to pneumonia. In our hospital, some children two to three months old and under one year old are infected with pneumonia.”	High incidence of disease for certain age groups	Age vulnerability	Perceived vulnerability
S09 “I will consider the vaccine for hand, foot, and mouth disease because my child’s aunt has a child who is one year older and had this disease.”	Children and other family members having had related diseases	Medical history	
S23 “I don’t think it’s necessary for him to get the vaccine when he grows up with improved immunity.”	Children’s own immunity and physical fitness	Immune quality	
S15 “During the COVID-19 epidemic, I was afraid of catching a cold; so, I got the vaccine.”	Surrounding environment increasing children’s vulnerability to diseases	Environmental vulnerability	
S06 “This vaccine can more or less protect him, of course not 100% but 80% for sure.”	Vaccination effectively preventing disease and improving immunity	Effect perception	Perceived efficacy
S21 “It does not truly matter whether we get the shot or not, but if we do, at least we’ll feel better.”	Vaccination providing inner comfort	Psychological comfort	
S14 “Because the flu is seasonal, [the vaccine] is only effective for this season, and then, you still have to get a new flu shot afterward.”	Vaccination continuously preventing disease	Protective strength	
S13 “It is good to have free vaccines, but you have to pay for other vaccines that you need but are not free.”	Existence of functions of non-NIP vaccines that cannot be replaced by those of NIP vaccines	Functional compensation	
S06 “Some vaccines are expensive, and ordinary families like ours may not be able to afford them.”	Parents’ judgment of the cost of non-NIP vaccination	Cost burden	Perceived cost
S20 “In addition, I’m still afraid that if something [bad] happens to my child after receiving the self-paid vaccine, it’s not worth it.”	Parents’ awareness of the possible negative impact of non-NIP vaccination	Adverse reaction	

*Perceived severity* refers to an individual’s subjective assessment of the seriousness and potential negative consequences of a specific threat, such as a disease, injury, or harmful event. In this study, it is people’s perception of the severity of the potential illness [22]. Analysis of the interview transcripts resulted in four subthemes under perceived severity, namely, physical harm, treatment consumption (cost of treatment of the subject diseases), psychological burden, and social consequences. Most interviewees expressed fear and concern about the clinical consequences of diseases prevented by non-NIP vaccines, and their perception of the cost of medical treatment and the consumption of nursing time due to the treatment of diseases promoted the establishment of vaccination intention. Maternal status played an important role in decision-making regarding the vaccination of children. In the interviews, most female interviewees indicated that they could not bear the psychological burden when their children were sick, strengthening their vaccination intention. The social consequences mentioned by the interviewees stood out as problems related to the children’s enrollment in school. When non-NIP vaccination is mandated by social institutions such as kindergartens and elementary schools or when the management of infectious diseases creates barriers to school enrollment and education, parents develop a “relatively passive” non-NIP vaccination initiative. The issue of school enrollment becomes an invisible force influencing vaccination.

*Perceived vulnerability* is the subjective judgment of the interviewees regarding the likelihood of their children developing a disease. The interview transcripts revealed four subthemes within this factor: age vulnerability, past medical history, immune quality (children's underlying immune status), and environmental vulnerability (sanitary condition of the village). Most interviewees adjusted their non-NIP vaccination intention according to the physical status of their children at different ages. For example, the interviewees frequently mentioned that children aged 1–2 years have a high incidence of HFM disease and that children under three years old are prone to pneumonia and therefore should receive the EV71 vaccine and 13-valent pneumococcal vaccine at these ages. The influence of “medical history” was often observed in families with two or more children, where a history of family members having been infected with a certain disease worked in conjunction with psychological burden to push parents to choose vaccination. Some parents had higher vaccination intentions when their children had low immune quality, and other parents believed that vaccinations would not work if children had low immune quality, thus leading to a low vaccination intention. This result indicates that some parents in rural areas lacked knowledge about health and children’s resistance and vaccine immunity. In addition, some interviewees were concerned about the environment where children gather (e.g., kindergartens) and believed that the relatively poor sanitary conditions in rural areas are more likely to expose children to germs, which rendered vaccination a possible solution. Some parents also mentioned that in the context of the COVID-19 pandemic, exposure to a hospital environment poses a higher risk of infection, and therefore, they would consider non-NIP vaccination to prevent the illness and avoid an infectious environment.

*Perceived efficacy* refers to an individual's belief in their ability to successfully execute a specific behavior or take recommended actions to achieve desired outcomes. In this study, it is the subjective perception of the benefits of non-NIP vaccination. Four subthemes, i.e., effect perception, psychological comfort, protective strength, and functional compensation (functions of non-NIP vaccines unreplaceable by NIP vaccine), were extracted from the interview transcripts. As preventive products, rural parents tend to believe that vaccines can prevent their children from suffering from certain diseases. Even if they did not fully understand the effect of non-NIP vaccines, the consumption value of “always spend money on something useful” still played a role for them in choosing vaccines. Other than the effect perception, the rural parents chose vaccinations due to psychological comfort. Vaccination intention increased when non-NIP vaccinations would save parents from blame and shame in case of sudden illness of their children. The perception of protective strength varied among the rural parents. Some interviewees thought highly of the prevention value of vaccines, while some mentioned a risk of becoming ill after vaccination and thus considered the protective strength of vaccines low and vaccination unnecessary, reflecting the fact that rural parents have not yet established the perception that vaccines are preventive rather than curative. Perceived functional compensation was another important factor influencing rural parents’ vaccination intention when substitutes for some non-NIP vaccines were not found in the NIP. Once a need for disease prevention was evident, the parents would perceive the nonsubstitutability of non-NIP vaccines, resulting in vaccination behavior.

*Perceived cost* refers to an individual's subjective evaluation of the negative aspects or barriers associated with adopting a particular behavior or taking specific actions. In this study, it is the subjective perception of the cost and consequences of non-NIP vaccinations. The choice to have children vaccinated is a benign protective behavior of parents that is associated with few risks. Two subthemes, i.e., cost burden and adverse reactions, were extracted in this theme. The commodity nature of non-NIP vaccines makes price an important moderator of vaccination intention. In economically underdeveloped rural areas, a higher perceived cost burden of receiving non-NIP vaccines corresponds to a lower vaccination intention. Regarding adverse reactions to vaccines, most of the interviewees held relatively objective perceptions, with only a small number of rural parents misled by rumors, and thought that vaccination could cause paralysis or death, increasing their fear of vaccinations and thus leading to vaccine hesitancy.

### Factors influencing vaccination intention from the social level

Vaccines are public health products. Social ecological theory [23, 24] emphasizes that an individual’s behavior is formed by interactions with the surrounding environment. Accordingly, health behavior is influenced by factors in an individual’s environment at the interpersonal, community, and public policy levels.

#### Social relations affect the vaccination attitude

Research reveals that social interactions among friends, relatives and neighbors at the interpersonal level may exert a significant impact on individuals’ attitudes and risk mitigation behaviors either through affecting people’s risk perception [25] or by nurturing interpersonal trust [26]. Rural parents have a relatively poor awareness and understanding of non-NIP vaccines; therefore, they subconsciously seek social relations to inquire about the vaccination status of other children. The non-NIP vaccination status of other children in the village has an enormous impact on rural parents’ vaccination intentions and behaviors by not only changing vaccination intentions but also reinforcing and shaping them. Most interviewees stated that after obtaining vaccine information from medical personnel or media channels, they would refer to the vaccination status of others around them before deciding whether to vaccinate.

“I would ask my relative as they also have children and will take their children’s health seriously. They told me that their children have never received non-NIP vaccines; so, I decided not to let my child take the shot either.” (S09).

#### Community institutions guarantee vaccination services

Social ecological models argue that health behaviors are an interplay of individual and environmental factors and have proposed multilevel frameworks [27]. Among them, community level factors in terms of delivery of community services, community physical environment and community capacity all play a role in affecting health outcomes [28]. In accordance with China’s vaccination policy, vaccination stations in each township in rural areas are responsible for vaccination services in their subordinate villages; the health center in each village does not provide vaccination services. As a result, rural parents must visit township hospitals regularly to receive vaccination services and must overcome the obstacles related to the time and transportation costs associated with traveling from remote villages to vaccination stations. In addition, the supply of some important non-NIP vaccines, that is, the accessibility of vaccination services, also becomes a key factor influencing vaccination intentions. As an interviewee said, “I wanted to get the 13-valent pneumococcal conjugate vaccine for my child, but there was no place to do it. Later, I went back to the vaccination station and asked again, and people there told me that I missed the vaccination time and that I would have to go all the way to Luoyang City to look for it.” (S05).

The vaccination station in each township not only provides vaccination services but is also responsible for vaccine-related health education. However, as several interviewees complained, “There are always a lot of people packed in such a tiny space, and doctors and nurses do not bother listening to you or answer your questions as there are many people waiting in line.” (e.g., S22, S07).

Taking Duan Village as an example, the vaccination station in the township health center has a total area of 50 square meters, covering NIP and non-NIP vaccination services for all children in 31 subordinate administrative villages. In contrast to urban children who can receive vaccinations at epidemic prevention stations, maternal and child care centers, and various community clinics, rural children have very limited options for obtaining non-NIP vaccination services. In this regard, vaccination intention and behavior are bound to be hindered if a vaccine supply is not in place and vaccination experiences are not improved in township vaccination stations.

#### Vaccine policies highlight the value of vaccinations.

Previous studies have stressed the importance of public policy in health promotion interventions from the social ecological perspective [28]. The establishment of NIP in China in 1978 and other vaccine-related policies have greatly promoted people’s vaccination awareness and behavior. At the public policy level, no mutually exclusive relationship is evident between non-NIP vaccination choices and the NIP. However, based on the interview data, NIP vaccines may influence the choice of non-NIP vaccines by parents to some extent. For example, the diseases prevented by combined vaccines such as the Diphtheria Tetanus Pertussis vaccine (DTaP) and the 5-in-1 Vaccine (DTaP-IPV-Hib) are partially covered by the vaccines in the NIP, and parents may not want to spend money on the non-NIP combined vaccines. In addition, substitutes can be found in the corresponding NIP for vaccines such as the AC meningococcal vaccine for the prevention of meningitis, which influences the choice of non-NIP vaccines by rural parents to some extent. As one interviewee mentioned, “Free ones have already been out there as a substitute; so, I don't feel the need to spend money to get the shot.” (S18) Moreover, some parents argued that those vaccines not included in the NIP were considered not important by the state and hence unnecessary; therefore, they chose not to receive any non-NIP vaccines.

In addition, NIP policies influence people’s vaccination behavior. Both the adjustment of national-level public policies and the expansion of immunization programs have impacts on actual non-NIP vaccination behavior. Strict control and supervision of the marketing and circulation of non-NIP vaccines, such as ensuring the quality of vaccine products, also provide positive guidance for non-NIP vaccinations. Government initiatives such as the promotion of mobile vaccination platforms, the implementation of health education activities, and efficient handling of vaccine rumors can also optimize vaccination trends in society and influence people’s non-NIP vaccination behavior.

### Construction of a non-NIP vaccination decision-making model

After obtaining the themes of influencing factors of non-NIP vaccination intention, this study constructed a structural model for the roles of these factors in the vaccination decision-making process based on the S–O-R model [29] of health behavior and decision-making. Specifically, rural parents’ non-NIP vaccination intention and behavior involve three important parts, i.e., stimulus (S), organism (O), and response (R).

#### Information sources as stimuli

The rural parents lacked the ability to learn and analyze vaccine information and knowledge, and their awareness of non-NIP vaccines mostly relied on information obtained through channels such as medical personnel, friends/relatives, media, the internet, and health education courses. The interviewees had higher trust in medical personnel than in other sources, which makes medical referrals the most important information source for non-NIP vaccination. When rural parents experienced difficulty in communicating with medical personnel, other information sources provided useful supplements. For example, the development of training courses, such as “Mummy Class,” and the Xiaodoumiao app played important roles in helping parents build effective vaccine awareness.

#### Establishment of vaccination intention by rural parents (organism)

After processing various pieces of information from different channels, rural parents evaluated the pros and cons of non-NIP vaccination. The interview data show that perceived severity, perceived vulnerability, perceived efficacy, and perceived cost are the four key individual-level factors influencing rural parents’ intention for non-NIP vaccination.

#### Occurrence and consolidation of vaccination behavior (response)

After an individual has established vaccination intention at the psychological level, actual vaccination behavior is also influenced by the surrounding environment. Social relations, community institutions, and vaccine policies all play roles in this process.

**Fig. 1:**
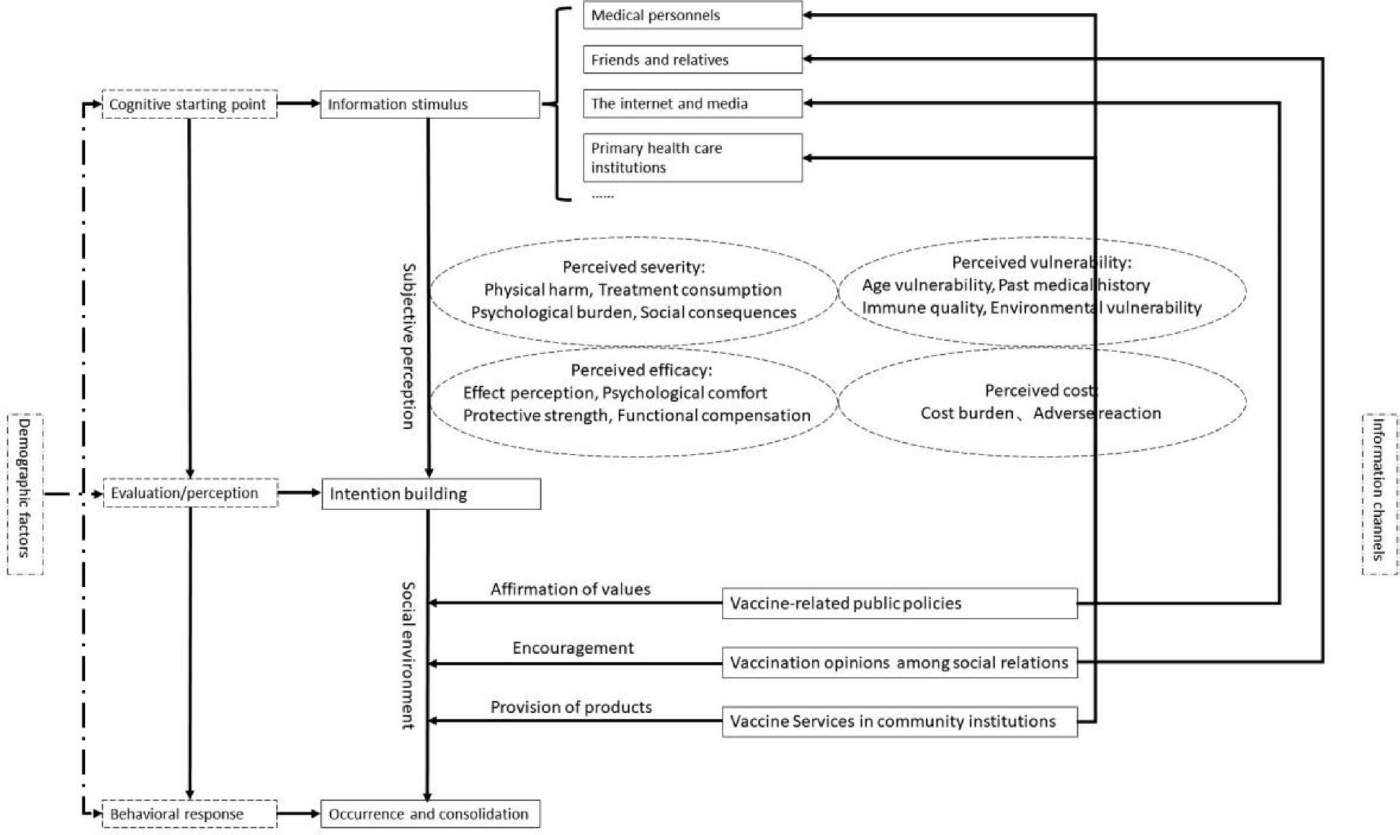
The non-NIP vaccination decision process model

Based on the analysis of the various factors involved in the vaccination behavior process, a model of the non-NIP vaccination decision of rural parents was constructed (see Fig. 1).

The above model shows the role of each factor in the vaccination decision process. Regarding vaccination awareness, medical personnel, friends and relatives, the internet and social media, and primary health care institutions play key roles as important information sources. Second, perceived severity, perceived vulnerability, perceived efficacy, and perceived cost are the four factors influencing individuals’ vaccination intention, with each factor consisting of several subthemes. In terms of specific behavioral responses, vaccination opinions among social relations, vaccine services in community institutions, and the support of vaccine-related public policies further strengthen and consolidate rural parents’ vaccination behavior. A structural framework was established through the Colaizzi seven-step data analysis method. The study concluded with basic structural validation using three copies of the original interview transcripts, and no new factors or other interference items emerged, thus ending the analysis.

## Discussion

This study investigated Chinese rural parents’ awareness and attitudes regarding non-NIP vaccines and found that vaccine awareness influenced attitudes toward vaccination and that information source channels played a positive role in shaping awareness, which is consistent with previous studies of the knowledge-attitude-practice (KAP) model. As predicted by the health belief model [22] and other health behavior change theories, rural parents’ non-NIP vaccination intention was influenced by such factors as perceived severity, perceived vulnerability, perceived efficacy, and perceived cost. These subjective assessments helped rural parents to evaluate the pros and cons of non-NIP vaccination. While the social level factors such as the vaccination opinions among rural social networks, the accessibility of health services and vaccine products, and the promotion of vaccination policies, further influenced the vaccination intention and behavioral decisions of rural parents, in addition to their subjective awareness.

Most existing studies focus on people’s vaccination intention on a single vaccine, such as HPV [30], the rotavirus [31] and Covid-19 vaccines [32], while this study examines the overall attitude toward the non-NIP vaccination. In addition, previous research investigates the vaccination intention of a certain group of people such as children, females or college students; few have paid attention to the rural–urban gap in non-NIP vaccination rate and the reasons behind it. The findings of the current study provide practical suggestions for promoting and expanding non-NIP vaccination in China as well as improving the health communication strategies targeted at rural parents.

### Practical applications

In terms of vaccine knowledge, the results indicate that vaccine education targeted at rural parents should focus on explaining the “type,” “efficacy,” and “adverse reactions” of non-NIP vaccines, including the effects of combined vaccines and different types of vaccines, which is consistent with the right of parents to be informed before making vaccination decisions. Factors that influence vaccination intentions, such as age vulnerability, social consequences, and the unreplaceable functions of non-NIP vaccines, should also be considered in designing vaccine education content. Additionally, regular public health education should be carried out in rural areas, not only to disseminate health knowledge but also to correct such misconceptions as “an illness is not serious unless it requires hospitalization,” “I would simply not get the shot if I may still get sick after vaccination,” and to stop rumors like “vaccination causes paralysis.”

Given the success of “Mummy Class” health courses, as shown in this study, we suggest that offline knowledge education activities be vigorously carried out to provide systematic training to parents of infants or to expectant mothers and that health education be conducted through various channels, such as online health communities and social media platforms, to avoid missing the best time for child vaccination, save time costs associated with communication with medical personnel, and improve the efficiency of rural vaccination services. In addition, considering the strong penetration of short video platforms in rural areas, vaccine education can be enriched through short videos and other means to support learning by parents.

This study also provides a reference for public health policy making. Both the accessibility and supply of non-NIP vaccines should be improved. For example, the coverage area of rural vaccination stations should be appropriately adjusted such that county hospitals near townships and village clinics with appropriate capacities can assume some of the vaccination service responsibilities of township vaccination stations. Appropriate policy support and economic assistance for rural non-NIP vaccination should also be considered. More importantly, the expansion of the current NIP in China should be prioritized. Important childhood vaccines, such as those for pneumonia, rotavirus, and Hib, should be included in the NIP. On the one hand, there is a gap between the importance of these vaccines and low vaccination rates in rural areas. On the other hand, these vaccines are relatively costly, and their supply is not controlled by the state and thus cannot be effectively guaranteed, thereby hindering the vaccination of rural children, both subjectively and objectively. In addition, considering the relatively poor environmental and sanitary conditions in rural areas, the costs of common vaccines such as those for varicella, HFM, and rabies can be subsidized via rural medical insurance plans to increase vaccination rates.

### Limitations and future research

This study is of great significance for building a structural model of the non-NIP vaccination decision-making process and providing practical implications to guide China’s vaccine policy in rural areas. Although the village selected for investigation can largely represent Chinese rural areas and is quite reliable in gauging the various factors that influence rural parents’ vaccination intention, it may not reflect China’s regional differences in terms of economic development, public policy, and health literacy. This study used purposive sampling and only interviewed parents with children aged 0–2 years, which may cause some errors and bias in the results. Since the interviewees of this study were rural parents and most had limited knowledge of vaccines, relevant explanations and interpretations were often needed when researchers communicated with them, which may cause a possible response bias from the researchers. Future studies could expand the scope of the research to include more rural areas for investigation and use random sampling to increase the generalizability of the findings.

In addition, the current study investigated non-NIP vaccines as a whole and did not consider the particularity and specificity of different non-NIP vaccines. Future studies can examine the vaccination intention of a particular vaccine of interest. Non-NIP vaccines are a social issue affecting children in society, and improvements in the NIP require a broader understanding of the needs of various groups in different regions.

## Data Availability

The datasets used and analyzed during the current study available from the corresponding author on reasonable request.
